# Long-term outcomes for 2-stage urethroplasty: an analysis of risk factors for urethral stricture recurrence

**DOI:** 10.1007/s00345-021-03676-8

**Published:** 2021-04-03

**Authors:** James R. Furr, Eric S. Wisenbaugh, Joel Gelman

**Affiliations:** 1 University of Oklahoma College of Medicine, 920 Stanton L. Young BLVD, WP 2140, Oklahoma City, OK 73104 USA; 2 Department of Urology, University of California, 333 City Blvd West, Suite 1240, IrvineOrange, CA 92868 USA

**Keywords:** Urethral stricture, Urethroplasty, Staged urethroplasty, Split thickness skin graft, Buccal graft

## Abstract

**Purpose:**

To report long-term results and patient reported outcomes of staged anterior urethroplasties, and isolate risk factors for recurrence.

**Methods:**

We reviewed urethroplasty database for all patients who underwent staged urethroplasty from 2000 to 2017. Follow-up included a cystoscopy 4 months after their 2nd stage to assess early success, and then annual follow-up thereafter with post-void residual and symptom assessment. Stricture characteristics, etiology and graft type were analyzed with regards to success.

**Results:**

Forty-nine patients were eligible for inclusion. The median stricture length was 7 cm (3–17 cm). The early success rate demonstrated by cystoscopy at 4 months was 100%. Long-term success was 96.4% in buccal graft (BMG) only patients; however, long-term success fell considerably to 53% in patients requiring any use split thickness skin graft (STSG) in the first stage. Median follow up time was 57 months (6–240 months). On analysis, age, increased stricture length and especially the use of STSG all appeared to be associated with late recurrence. The recurrence group had longer stricture length and were more likely to be panurethral. All recurrences occurred after the initial 4-month cystoscopy with a median time to recurrence of 78 months.

**Conclusion:**

Staged repairs that are amenable to BMG-only repairs have high long-term success rates. Increasing stricture length and the addition of split-thickness skin graft were associated with lower success rate in staged urethral reconstruction. Patients requiring staged repairs often experience recurrence in a very delayed fashion reinforcing the need for close, long-term follow up.

## Introduction

Staged urethroplasties are becoming less frequent as many long, complex urethral strictures are now being repaired in a single stage [1]. However, certain strictures are at times not amenable to single stage repairs due to a corpus spongiosum or urethral plate that is unsuitable for augmentation. As a consequence, staged repairs are still commonly performed for strictures as a result of failed prior hypospadias surgery, highly complex strictures that have been treated with prior open reconstruction, and select complex cases of lichen sclerosus (LS) also known as Balanitis Xerotica Obliterans (BXO).

It is now clearly established that genital skin is not recommended for the treatment of urethral strictures associated with LS when used as flap or grafts [2]. Despite early success, this change in clinical practice away from the use of genital skin occured because of the very high late failure rate that was observed when there was long-term follow-up of up to 10 years [3, 4]. The fact that substitution urethroplasty including staged repairs can in certain cases, be associated with a high late failure rate indicates the importance of long-term outcome assessment.

Schrieter et al. first described the use of split thickness skin grafts (STSG) including extra-genital grafts for a 1st stage operation [5, 6]. Buccal mucosa is now the graft material of choice for single-stage repairs due to excellent overall success rates, and is now being used with increased frequency for staged repairs [1, 7]. However, the quantity of available oral mucosa is limited. Sufficient graft material must be harvested to insure tubularization to a normal caliber during the second stage repair along the entire area of stricture in complex, panurethral disease. One advantage of the use of STSG from the thigh is the ease and speed of harvesting sufficient graft material. While patients with stricture disease that requires a staged approach to urethroplasty are a relative minority, they often represent some of the most challenging patients in urethral reconstruction.

Although the use of extra-genital STSG and buccal mucosa grafts (BMG) are currently favored for staged urethroplasty, there are gaps in the current published literature. The reported short-term success rate is generally high. However, the limitations of the current literature include relatively short follow-up, the inclusion of multiple different surgical techniques without separate assessment of a staged-urethroplasty outcomes, or the lack of a clearly defined follow-up protocol to assess post-operative anatomic success and/or long-term results [8–10]. We aim to present an analysis of our series of staged urethroplasties using BMGs and/or STGT harvested from the thigh to include early assessment of anatomic success and long-term follow-up with patient reported outcome measures (PROMs). It was hypothesized that with longer follow-up, the late recurrence rate would increase, and that the inclusion of skin grafts could represent a risk factor for late failures.

## Materials and methods

### Patient selection and preoperative evaluation

With IRB approval, a prospectively maintained urethroplasty database was queried isolating all patients who underwent staged repair from 2000 to 2017. Pre-operatively, all patients underwent a flexible urethroscopy, a retrograde urethrogram, and a voiding cystourethrogram. Candidates for staged urethroplasty were selected in accordance with treatment algorithms previously described [11]. In short, patients who underwent staged repairs generally lacked an intact corpus spongiosum or urethral plate that could be adequately mobilized and augmented with a substitution 1-stage urethroplasty. As such, the study included hypospadias surgery failures, prior reconstructive failures, and certain patients with complex lichen sclerosus that extended into the fossa navicularis. Isolated meatal and fossa strictures were excluded. Additionally, patients were excluded if any portion of their repair was performed in a single stage, or if they elected to not pursue a second stage urethroplasty.

### Operative technique

The 1st stage was performed by making a ventral urethrotomy through the stricture, ensuring that the urethra proximally was non-hirsute and widely patent (ideally 30Fr). We accomplished this by bougie calibration and cystoscopy once the entire stricture had been incised. The urethral plate was typically left in the midline, although it was excised if it did not contribute significantly to urethral width or was hirsute. The goal was to transfer enough graft to allow the entire area of the recipient bed to measure slightly more than 3 cm in width, to allow for a 30Fr urethra when tubularized during the 2nd stage surgery. Early in our series, we performed staged repairs on two patients using STSG alone harvested from the thigh. As BMGs gained popularity, we began to favor the inclusion of buccal grafts along with or instead of STSG. However, with longer complex strictures that required extensive tissue transfer, STSG was used when bilateral buccal grafts did not provide sufficient graft material.

**Fig. 1:**
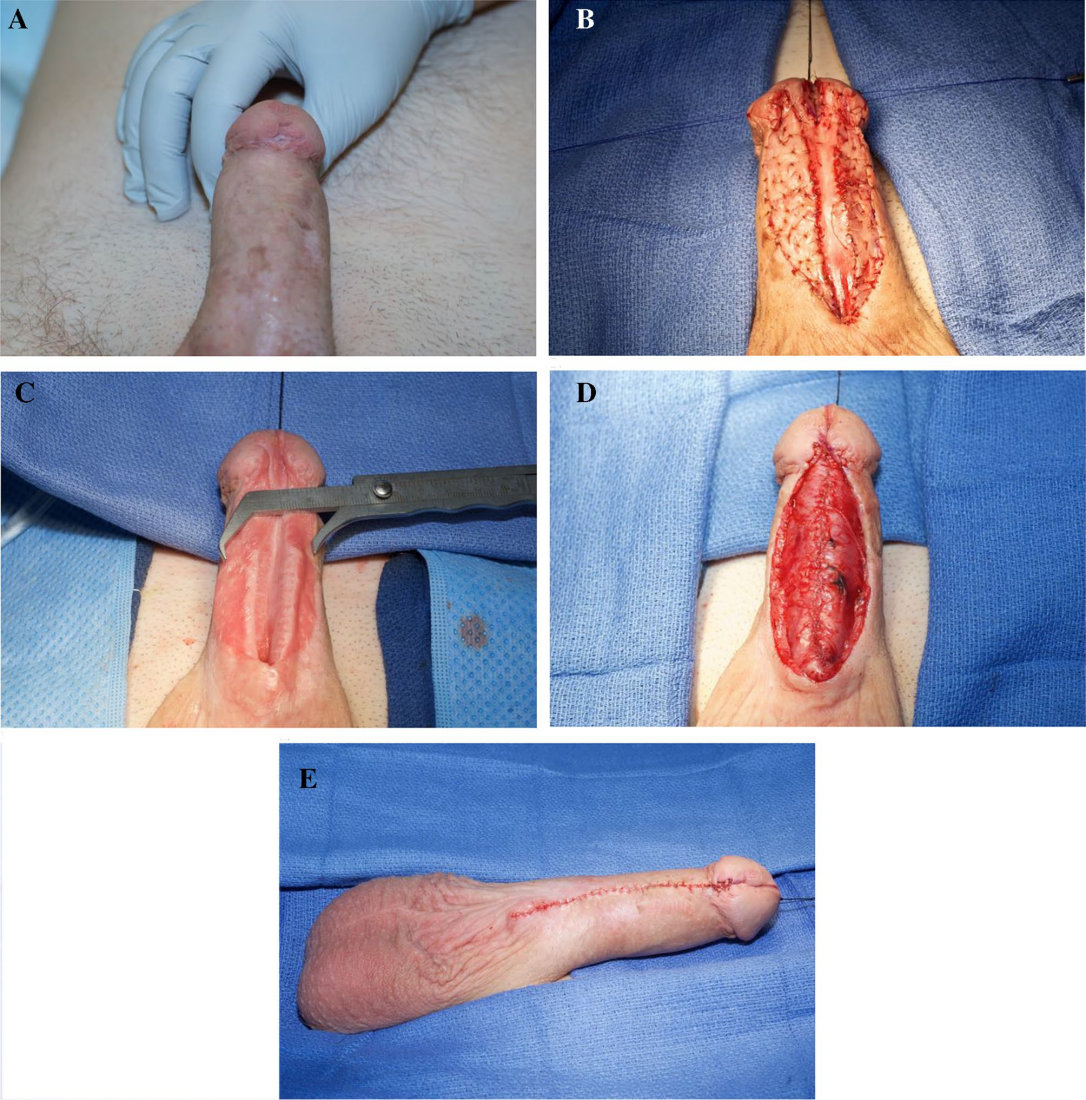
**a** Patient with a long penile urethral stricture and history of hypospadias. **b** The ventral urethrotomy has been made and BMG fixated to either side of the urethral plate. **c** Demonstration of 3 cm width to the healed urethral plate to allow for a 30Fr caliber lumen. D&E) Closure is accomplished in multiple layers

The 2nd stage, performed at least 4 months later, involved tubularization of the neo-urethra followed by a multi-layer closure. We attempt to bring the meatus as distally as possible, but did not risk sacrificing caliber to bring it to the most distal aspect of the glans. Pictures illustrating the different surgical steps are displayed in Fig. 1.

### Post-operative care

A bolster was applied at the end of the 1st stage operation and kept in place for 5 days. The bolster consisted of xeroform gauze directly applied to the graft with overlying dacron wool soaked in mineral oil, all secured with sutures to skin; the urethral stenting catheter was removed approximately 10–14 days after surgery. Following bolster removal, patients were asked to keep xeroform gauze on the graft for another 14 days.

Patients were assessed 4 months after first-stage repair to confirm adequate graft for tubularization, wide patency of the ventrally displaced meatus, and a wide caliber of the urethra proximal to the meatus with flexible cystoscopy. After the 2nd stage procedure, the urethral stenting catheter was removed at 3 weeks, and a voiding cystourethrogram was obtained. Our follow-up protocol after the 2nd stage included distal urethral calibration with bougie-a-boules and cystoscopy at 4 months to ensure early success, and then annual follow-up thereafter with a post-void residual and symptom assessment to assess long-term success. At each annual follow-up, office staff collected patient reported outcomes such as the International Prostate Symptom Score (IPSS) and the sexual health inventory for men (SHIM). We also obtained urethroplasty specific PROMs which included assessment of delay in starting stream (0 “never”—4 “all the time), rates of post void dribbling (0 “never” —4 “all the time), patient satisfaction, and whether or not patients would elect to have their surgery again [12].

### Data analysis

Patient characteristics such as age, stricture length, comorbid conditions such as LS or hypospadias, and the use of BMG and/or STSG were isolated The primary outcome was urethral patency, with a particular focus on the long-term success rate. Other endpoints assessed were patient reported outcomes (PROMS). Early anatomic success was defined as the ability to easily pass a 16Fr cystoscope at the 4-month cystoscopy after the 2nd stage repair (correlating to early, technical success). Long-term functional success was defined as either an absence of lower urinary tract symptoms based on history and IPSS and other urethroplasty-specific and satisfaction-related PROMs, or confirmation of continued wide urethral patency on repeat cystoscopy if there was development of obstructive voiding symptoms, elevated residuals or urinary tract infections. We did not have defined "triggers" such as a certain numerical change in a symptom score as an indication to perform a follow-up cystoscopy. The decision was often related a reported adverse change in symptoms such as a decreased force of stream, and/or a decrease in satisfaction due to an adverse voiding symptom change. Length of follow-up was determined by the interval between the date of surgery and the date of most recent evaluation, not the time between the date of surgery and manuscript submission. Both mean and median based data were incorporated into our analysis for continuous variables, specifically age, stricture length and follow-up. Non-parametric mann–whitney *U*, chi-squared, and Fisher’s exact tests (for categorical data) were used when appropriate, and a *t*-test was also included in analysis for continuous, mean-based variables. Analysis was performed with StataSE (College Station, TX). Statistical significance was set at *p* < 0.05.

## Results

A total of 85 patients underwent 1st stage repairs during the study period, of which 57 patients underwent both 1st and 2nd stage repairs. No patient required a revision surgery subsequent to the 1st stage repair to add additional graft to insure sufficient width (30 + mm) for tubularization. One young patient who had a pan-urethral stricture after failed proximal hypospadias repairs and underwent 1st stage repair with STSG and BMG did undergo a procedure to add buccal mucosa to the fossa navicularis prior to the second stage repair. The objective was to widen the fossa with a mucosal graft that would provide a more normal appearing urethral opening after tubularization.

In total, 49 were compliant with follow-up and eligible for inclusion. Two of these 49 patients who were compliant with long-term follow-up did not return for their 4-month cystoscopy, and they were included only in the long-term functional outcome analysis. There were 5 patients who underwent cystoscopy at 4 months, but did not have 12 or more months of follow-up after surgery. They were excluded from the long-term analysis.

Of included patients, 37 (77%) had a diagnosis of hypospadias, 7 (15%) had LS, 2 had both, and three had very complex, recurrent strictures in the absence of either diagnosis. The vast majority (80%) had a history of prior failed urethroplasty. The median stricture length was 7 cm (range 3–17 cm) with a median follow-up of 57 months (range 6–240 months). Median time between first and second stages was 6 months. Patient demographics for the staged urethroplasty cohort are summarized in Table 1.

**Table 1:** Patient Demographics and Outcomes of Staged Urethroplasties

Number of patients	49
Median age (years)	37 (range 16–75)
Mean age (years)	37.6 (95% CI 33.2–42.0)
Diagnosis (%)	
Hypospadias	37 (75.5%)
LS	7 (14.3%)
Both	2 (4.1%)
Neither/Recurrent Stricture Disease	3 (6.1%)
Location	
Meatal/Fossa extending to Penile	27 (55%)
Penobulbar/Panurethral	22 (45%)
Prior failed reconstruction (%)	39 (80%)
Median stricture length (cm)	7 (range 3–17)
Mean stricture length (cm)	7.5 (95% CI 6.3–8.6)
Median post op IPSS	4 (range 0–13)
Median post op SHIM	20 (range 0–25)
% Worsening sex	17.5%
Median follow-up	57 months (range 6–250)
Mean follow-up	63.7 months (95% CI 54.2–83.6)
Median time between stages	6 months (range: 4–75)
Median delay in starting stream (0 “never”—4 “all the time”)	0
Median post void dribbling (0 “never”—4 “all the time”)	1
Complications (%)	4 (10%)
Satisfaction rate	86%
% Would have surgery again	100%
Early technical success	100%

STSG and BMG was used in the first stage in 15 patients, with 2 patients undergoing STSG-only first stages early on in our series. This is compared to 32 patients whose 1st stage was grafted with BMG only. The STSG group trended toward longer median stricture length (8 cm vs 5 cm). However, no differences were noted in rate of LS or hypospadias in each group as can be seen in Table 2.

**Table 2:** Comparative Analysis of Outcomes

	Analysis of recurrence			Comparison of BMG vs. STSG outcomes		
	Success (*n* = 40)	Recurrence (*n* = 9)	*P*-values	STSG in any portion (*n* = 17)	BMG only (*n* = 32)	*P*-values
Median Age (years)	34	48	**0.02** (Mann–Whitney *U*)	37	35	0.93
Mean Age (years)	35.3	47.9	**0.02** (*T*-test)	38.2	37.4	0.93
Etiology (*n* =)			0.515			0.32
% Hypospadias	77.5%	66.7%		64.7%	81.25%	
% LS	17.5%	22.2%		29.4%	12.5%	
% Only Multiple Failed Reconstruction	5.0%	11.1%		5.9%	6.25%	
Median Stricture Length (cm)	5.0	8.0	0.05 (Mann–Whitney *U*)	10	5	** < 0.001**
Mean Stricture Length (cm)	6.8	10.0	**0.02** (*T*-test)	10.2	5.9	**0.003**
Location			0.12			** < 0.001**
% Meatal/Fossa extending to Penile (*n* =)	60.0% (24)	33.3% (3)		19% (3)	75% (24)	
%Bulbopenile/Panurethral (*n* =)	40.0% (16)	66.7% (6)		81% (14)	25% (8)	
Median Post Op IPSS	3	5	0.25	4	3	0.56
Median Delay in Starting Stream (0 “never”—4 “all the time”)	0	2	0.18	0	1	0.35
Median Post Void Dribbling (0 “never” -4 “all the time”)	1	1	0.26	1	1	0.94
Median Post Op SHIM	20	15	0.55	13	15	0.69
% Worsening Sex (*n* =)	17.2% (6)	20.0% (1)	0.64	17.8% (5)	16.7% (2)	0.654
% with STSG (*n* =)	22.5% (9)	88.9% (9)	** < 0.001**			
Long-term Success (> 1 year)^a^				53%	96.4%	** < 0.001**
Median Length of Follow-up (months)	46.03	127.9	**0.001** (Mann–Whitney *U*)	79	45	**0.02**
Mean length of follow-up (months)	55.8	127.9	** < 0.001** (*T*-test)	94.9	55.5	**0.027**
Satisfaction	86%	50%	0.06	73%	86%	0.275

Bold values signify outcomes that are statistically significant

^a^Patients without at least year-long follow up excluded

The early technical success rate for all patients in both groups demonstrated by cystoscopy at 4 months following 2^nd^ stage reconstruction was 100% (Table 1). We also noted a 96.4% long-term success in BMG only patients with one late recurrence isolated to the distal cm of the urethra; however, long-term success fell considerably to 53% in patients requiring any use STSG in the first stage, which included late recurrences in both of the men whose first-stage consisted only of STSG. Only patients with follow-up time of > 1 year were included in the analysis of long-term follow-up, with four patients being excluded. Mean time to recurrence was 95 months, and median time was 78 months (range 22–164 months). Complications (outside of stricture recurrence) were experienced in 10% (*n* = 5), which included two urethrocutaneous fistulas, one patient with significant meatal redundancy of buccal graft after second stage requiring revision, and two urinary tract infections. No patient had a donor site complication.

On univariate analysis, age, the use of STSG and increased stricture length all appeared to be associated with repair failure. The recurrence group trended towards significantly longer length and were more likely to be panurethral (Table 2). With regards to questionnaire data and PROMs, the cohort as a whole had favorable post op IPSS and SHIM results (Table 1). Additionally, the rates of post void dribbling and delay in starting stream was low, with median answers of < 1, correlating between “never” and “occasionally” on the questioniarre. While not statistically significant, these outcomes tended to be worse in patients who had STSG and who had recurrences (Table 2). Overall satisfaction rate was 86%, and 100% of patients stated they would have the surgery again. However, four patients who recurred declined to answer the satisfaction portion of the PROM questionnaire, and could likely be assumed to be unsatisfied. Of the patients that were unsatified, three stated that their symptoms did not improve, one patient was bothered by post void dribbling. Another complained of recurrent infections despite anatomic success, and one last patient had bother associated with decreased sensation. Of the nine recurrences, eight underwent retreatment, and one continued observation (Table 3).

**Table 3:** Summary of recurrences

	Graft	Etiology	Age (Years)	Recurrence time (Month)	Stricture length (cm)*	Complications	Management
1	STSG only	LS	54	22	17		Extended meatotomy
2	STSG only	Failed flap	33	96	7	Fistula	Repeat buccal urethroplasty
3	Buccal-STSG	Hypospadias	48	162	6		Perineal Urethrostomy
4	Buccal-STSG	Hypospadias	44	120	15		Perineal Urethrostomy
5	Buccal-STSG	Hypospadias	54	78	8		Repeat urethroplasty
6	Buccal-STSG	Hypospadias	63	26	15		DVIU with self-dilations
7	Buccal-STSG	Hypospadias	49	29	6		Observation
8	Buccal-STSG	Hypospadias	69	24	11		Perineal Urethrostomy
9	Buccal	LS	27	155	4.5		Extended meatotomy

*Stricture length refers to original stricture length

## Discussion

### Definitions of early and long-term urethroplasty success

While it is ideal to repair a urethral stricture with one operation, those with highly complex strictures following multiple repairs, prior hypospadias or LS often are often best suited to a staged approach. The reported success of staged repairs has varied considerably in published reports, and a major factor in this wide variation may be the different definitions of success along with differences in the length of follow-up [13]. Anatomic urethroplasty success is often defined as wide patency confirmed with cystoscopy 4–12 months after surgery [14]. This is a reflection of early and technical success. Another outcome measure is functional success that can be defined as an absence of symptoms, peak flow rates of at least 12–15 ml/s, or if there is no need for future stricture treatment. There is the potential for these definitions of success to underestimate recurrences. For example, it is quite possible that a man with a symptomatic recurrent stricture did not elect to pursue further treatment. In addition, it has been shown that the use of International Prostate Symptom Score (IPSS) alone is an inadequate tool to screen for stricture recurrence [15]. Moreover, changes in peak flow rate may be associated with recurrent strictures rather than wide urethral patency [16]. The most appropriate definition of a successful urethroplasty should incorporate both objective (anatomic) and subjective (functional) outcome measures to include specific patient reported outcome measures (PROMs), including quality of life and satisfaction assessment, and all of these definitions were incorporated into our study protocol [17].

Although a particular urethroplasty can associated with both early anatomic and early functional success using the above definitions, the durability of an initially successful repair is of major importance when assessing the utility and value of a substitution technique. In contrast to urethral reconstruction with excision and primary anastomosis, substitution urethroplasty can be especially prone to late recurrences after 5–10 + years [18]. A striking example of a surgery that is associated with good early success, but a very high late failure rate, is substitution urethroplasty using genital skin for the treatment of LS strictures. The use of genital skin as flaps or grafts in 1 or staged repair was once commonly performed, and was associated with good early success. However, when it was reported that with up to 10 years of follow-up, the recurrence rate approached 100%, this technique was abandoned [3, 4, 19]. In contrast, comparisons supporting BMG use over other extra-genital graft material in staged repairs is scant.

### The use of staged repairs for urethroplasty

The first description of the use of staged repairs using mesh foreskin and STSG grafts was by Schreiter [5, 6]. The reported early success rate was 99% in 96 patients with the majority of patients having at least 2 years of follow-up [6]. This technique was subsequently adopted by others. In 1997, Webster's group reported an 80% success rate in 20 men with a median follow-up of 38 months using STSG from the thigh in the vast majority of the cases [20]. This was followed by reports of the use of extra-genital grafts using buccal mucosa and postauricular skin to treat LS strictures with high success rate of 94% at a mean follow-up of 3 years. None of the above studies from the older literature included a protocol to assess urethral patency using anatomic or functional PROMs.

When 2-stage buccal mucosa urethroplasty was initially reported, the success rate was 93% with a median follow-up of 18 months [21]. As buccal mucosa became more favored as a graft material for substitution urethroplasty, numerous reconstructive Urologists began to report their results with buccal grafts. In a multi-institutional evaluation of all treatments for long-segment strictures by Warner et. al, the patients who were treated with skin grafts as opposed to BMGs had a higher recurrence rate [22]. A limitation of this comparison was a mean follow-up of only 20 months, and it was not stated if the skin grafts were genital or extra-genital. More recently, multiple studies have compared the performance of STSG with BMG directly with regards to staged repairs. These reports have shown comparable success rates between STSG and BMG groups [23–25]. However, two studies by Pfalzgraf et al. and Kluth et al. are limited by follow-ups of less than 1 year [24, 25]. In a another study by Pfalzgraf et al., follow-up approached 3 years with comparable quality of life measures, though overall patient satisfaction was lower in the STSG group versus the BMG group (83% versus 93.7%) [23].

Our results also indicate a very high early success rate defined by anatomic confirmation of wide patency of the repair 4 months after the 2nd stage repair using cystoscopy. We had no early recurrences of stricture in any patient. Moreover, our long-term functional outcome analysis revealed durable results when buccal mucosa was the only graft material used with only one late distal recurrence. However, in contrast to our high success rate for staged repairs using BMGs, the late recurrence rate was only 53% when any STSG was included in the repair. Our data determined that, in addition to the use of any STSG, increased stricture length, and to a minor extent, age were associated with recurrence. It is unclear if age is in and of itself is a risk factor for failure, but given the relatively low number of failures, subset analysis with a focus on age cannot be performed. Of the nine patients that had late recurrences, only two had a diagnosis of LS, suggesting that graft material and perhaps stricture length may represent more significant risk factors than stricture etiology. However, in some patients who presented with a history of multiple failed surgeries for stricture related or not related to hypospadias repair, the scarring and recurrence may have been related to associated LS. We did not routinely biopsy patient who underwent staged repair.

### Stricture length and the use of STSG as risk factors for late recurrence

Stricture length has been implicated in contemporary studies as an independent risk factor for repair failure for single-stage urethroplasties [26, 27]. These results also confirm those data reported by Selim et al., who also found length to be a risk factor for failure in staged repairs [28]. Kozinn et al. also reported a large series of staged urethroplasties (all with BMG-only) and reported first stage failures with mean stricture length of 11.7 cm, and second stage failures with mean stricture length of 14 cm [29]. Based on such findings, there appears to be a clear association with increased stricture length and failure.

The most striking finding was the major decline in long-term success rates when extra-genital STSG harvested from the thigh was used exclusively or included as a tissue transfer graft material compared to staged repairs where BMG alone was used. However, we want to stress the limitations of our study. The patients who underwent repair that included STSG tended to have longer and more complex strictures which could represent a risk factor for failure. However, although four of the patients who had recurrences after having both STSG and BMG placed had > 10 cm strictures, the other, the other four had relatively short (4.5–8 cm) strictures, which could have likely been treated with only BMG. We were less aggressive in using buccal mucosa alone earlier in our series. Therefore, late recurrences in these patients whose repairs included the use of STSG were not limited to only patients who had very long strictures. This is a qualitative observation as opposed to a quantitative analysis, and further research should be dedicated to determining what extent graft material plays a role in the durability of repairs for long, complex strictures.

In addition, the length of follow-up in the group that had repairs that included the use of STSG was considerably longer. In part, this was due to a shift towards a more aggressive use of oral mucosa later in our series, but certainly a longer follow-up is often associated with a higher late recurrence rate. Our failures all occurred late, with a median time to recurrence of 78 months (Table 4). This highlights the importance of the long-term follow-up, as prior studies with significantly shorter follow-up would not have captured any of these late recurrences. Therefore, although the mean follow-up in our BMG only patients was a mean of 55 months (Range 6–140 months), with longer follow-up, it is possible that we will see late recurrences in this group.

Given that repairs requiring BMG only had a 93% long-term success rate with a drop off to 53% when STSG was added, despite the limitation of our study, this observation indicates that STSG may be an inferior graft material for staged urethroplasties. This finding also appears consistent with data previously suggested by Bracka et al. [6] and Warner et al. [22].

If the goal is to avoid the use of not only genital but also extra genital skin for very long narrow caliber pan-urethral strictures, there is the issue of limited graft material that can be obtained from bilateral buccal graft harvests. Other types of oral mucosa such as lower lip and lingual grafts have been harvested and used in single-stage procedures with good success and low complication rates [30–32]. These grafts have very similar characteristics to BMG and could possibly offer higher success rates if used in lieu of STSGs, but the morbidity of harvesting bilateral BMGs and lingual and/or lower lip grafts has not been well studied. Given our findings, we intend to avoid STSG in the future, and will harvest lower lip grafts in addition to bilateral buccal grafts when additional graft material is needed.

A novel aspect of our presented data, in addition to the long-term follow-up, is the inclusion of PROMs with validated questionnaires in patients undergoing staged urethroplasty. Pre operative data were not routinely collected early on in the series with limited pre-operative and post-operative comparisons. However, our data do show that successful staged repairs result in very adequate post-operative IPSS, low complication, and high satisfaction rates. These results compare well to that which is reported for successful one-stage substitution repairs and excision and primary anastomosis [33, 34].

## Conclusion

Staged repairs that are amenable to BMG-only repairs have high long-term success rates. Increasing stricture length and the use of split-thickness skin graft resulted in lower success rate in staged urethral reconstruction. These patients tended to have longer, complex strictures, but perhaps most importantly, recurrences typically occur in a delayed fashion reinforcing the need for close, long-term follow-up.
